# Novel single-domain antibodies against the EGFR domain III epitope exhibit the anti-tumor effect

**DOI:** 10.1186/s12967-020-02538-y

**Published:** 2020-10-06

**Authors:** Tao Chen, Xue Liu, Haifeng Hong, Henry Wei

**Affiliations:** Department of Cell Biology and Institute of Biomedicine, National Engineering Research Center of Genetic Medicine, Guangdong Provincial Key Laboratory of Bioengineering Medicine, College of Life Science and Technology, Jinan University, Guangzhou, 510632 Guangdong China

**Keywords:** EGFR, Cancer, Antibody, Single-domain antibody, Antibody phage library

## Abstract

**Background:**

Monoclonal antibodies (mAbs) have been used for cancer therapy. They are large and have some disadvantages limiting their use. Smaller antibody fragments are needed as their alternatives. A fully human single-domain antibody (sdAb) has a small size of only 15 kDa and consists of only the variable domain of the human antibody heavy chain (VH). It has no immunogenicity. It can easily penetrate into tumor tissues, target an epitope inaccessible to mAb and be manufactured in bacteria for a low cost. Epidermal growth factor receptor (EGFR) is over-expressed in many cancer cells and is a good target for cancer therapy.

**Methods:**

The EGFR protein fragment located on the EGFR extracellular domain III was chosen to screen a human sdAb library. Five human anti-EGFR sdAbs were identified. Their specific binding to EGFR was confirmed by ELISA, Western blotting and flow cytometry. Their anti-tumor effects were tested.

**Results:**

Five novel fully human anti-EGFR sdAbs were isolated. They specifically bound to EGFR, not to the seven unrelated proteins as negative controls. They also bound to the three different human cancer cell lines, but not to the two cell lines as negative controls. They inhibited cell proliferation, migration and invasion and increased apoptosis of these three cancer cell lines. Two of them were tested for their anti-tumor effect in vivo and showed the anti-tumor activity in a mouse xenograft model for human lung cancer. Immunohistochemical staining of xenograft tumors also showed that their anti-tumor effects were associated with the inhibition of cancer cell proliferation and the promotion of cancer cell apoptosis.

**Conclusions:**

This study clearly demonstrated that the anti-EGFR sdAbs could inhibit cancer cell growth in vitro and tumor growth in vivo. They could be potential therapeutics for the treatment of different human cancers.

## Background

Epidermal growth factor receptor (EGFR) family comprises the four homologous members: EGFR or HER1, also known as ERBB1, HER2 or ERBB2, HER3 or ERBB3 and HER4 or ERBB4 [1]. Each member consists of an extracellular domain (ECD), a transmembrane domain and an intracellular domain (ICD) [2]. EGFR contains an extracellular domain (EGFR-ECD) of 620 amino acids, a transmembrane domain of 23 amino acids and an intracellular domain (EGFR-ICD) of 501 amino acids. EGFR-ECD comprises four domains, and the domains I and III include EGF binding sites. The EGFR-ICD includes a tyrosine kinase site. After EGF binds to EGFR, EGFR dimmer is formed and induces intracellular tyrosine phosphorylation, leading to the activation of the EGF/EGFR signaling pathway [3]. Studies indicate that the EGF/EGFR signaling pathway interacts with EGFR-ERK, EGFR-STAT3 and EGFR-mTOR signaling pathways [4]. These pathways are associated with tumor cell proliferation, vitality, migration and invasion. They are also associated with the angiogenesis in the tumor microenvironment, tumorigenesis and tumor progression and metastasis [5, 6]. According to the previous reports, EGFR and HER2 are expressed mostly at a low level in all normal cells, while HER3 and HER4 are expressed at a moderate to high level in all normal cells. EGFR over-expression in cancer cells is closely associated with poor clinical prognosis, reduced survival rate and more aggressive phenotype [7]. EGFR over-expression was shown in several solid tumors, such as lung, breast and prostate cancers [8–10]. Therefore, EGFR may serve as an attractive target for cancer therapy of different cancers [11].

Monoclonal antibodies (mAbs) have become powerful therapeutics for cancer therapy. Trastuzumab is an anti-EGFR mAb approved for the treatment of breast cancer. Cetuximab is another mAb targeting EGFR and was approved for the treatment of metastatic colorectal cancer, metastatic non-small cell lung cancer and head and neck cancer. However, mAbs used for cancer therapy have many disadvantages. Mouse-derived mAbs can result in rapid antibody clearance, loss of efficacy or hypersensitivity reactions in cancer patients [12]. Along with the development of genetic engineering, chimeric antibodies or humanized antibodies were developed and reduced only some of their antigenicity [13]. MAbs are difficult to penetrate into solid tumors due to their large molecular weight and expensive to be manufactured with mammalian cells [14, 15]. Thus, small size antibodies are needed to replace the conventional mouse-derived mAbs for cancer therapy. Single-chain variable fragment (scFv) is a small-size antibody of about 40 kDa, consisting of only the variable domains of the mAb heavy (VH) and light chains (VL). For example, ScFv-1171 is a derivative of mAb panitumumab and can bind to HER1 and induced apoptosis in rhabdomyosarcoma cells [16]. Human single-domain antibody (sdAb) of about 15 kDa consists of only the variable domain of human antibody heavy chain and has no immunogenicity [17, 18]. It has good tissue and tumor penetration [19]. It can access an epitope inaccessible to the large-size conventional mAb [20, 21]. Moreover, human sdAb can be easily manufactured in bacteria at a very low cost. Fully human sdAbs are less stable and soluble than the natural camelid heavy chain-only antibodies due to the lack of conserved framework region residues. Studies have significantly increased the sdAb stability and solubility by constructing synthetic human sdAb library from reference to the natural camelid heavy chain-only antibodies [22].

In this study, an EGFR protein fragment located at its extracellular domain was chosen for screening a fully human sdAb library. Five human anti-EGFR sdAbs were identified and tested for their effect on the three different cancer cell lines. Two of them were tested for their effect on a mouse cancer model. They showed potent anti-tumor activity in vitro and in vivo and can potentially become good candidates for the treatment of various cancers.

## Materials and methods

### Reagents and cell culture

Anti-M13-horseradish peroxidase conjugate (anti-M13-HRP) was purchased from Sino Biological (Beijing, China). Protein A-HRP was purchased from Thermo Fisher (Waltham, MA, USA). Expression vector pET22b (+), *E. coli* DH5a and BL21 (DE3) were purchased from Novagen (EMD Millipore, Madison, WI, USA). Isopropyl-β-d-thiogalactopyranoside (IPTG), phenylmethylsulfonyl fluoride (PMSF) and annexin V/PI apoptosis detection kit were purchased from Sangon Biotech (Shanghai, China). Nickel nitrilotriacetic acid (Ni^+^ -NTA) resin was purchased from Sevensea Biotech (Shanghai, China). Dimethyl sulfoxide (DMSO) and 3-(4,5-Dimethylthiazol-2-yl)-2,5 diphenyltetrazolium bromide (MTT) were purchased from Sigma Aldrich (St. Louis, MO, USA). Matrigel and transwell chambers were purchased from BD Biosciences (San Jose, CA, USA). Cis-platinum was purchased from the pharmacy of the first affiliated hospital of Jinan University (Guangzhou, China).

Human cancer cell lines (A549, DU145 and MCF-7) were purchased from American Type Culture Collection (ATCC, Manassas, VA, USA). A549 and DU145 cells were cultured in RPMI 1640 medium (Invitrogen, Carlsbad, CA, USA) supplemented with 10% fetal bovine serum (FBS, Invitrogen). MCF-7 cells were cultured in DMEM medium (Invitrogen) supplemented with 10% FBS. Cells were cultured at 37 °C in a humidified incubator containing 5% CO_2_.

### Screening for anti-EGFR sdAbs by phage display

Human domain antibody library (DAb) was purchased from Source BioScience (Nottingham, UK). A single human V3-23/D47 VH framework was used for the construction of the fully human sdAb phage-display library with diversity introduced in the antigen-binding site. The diversified hypervariable region in complementarity determining region 1 (CDR 1), CDR 2 and CDR 3 included H27-H33, H35, H50, H52-H54, H94, H95-H100 (a–k), H101 and H102. The library has 3 × 10^9^ sdAb clones in an ampicillin resistance phagemid vector pR2 containing MYC and VSV tags. Phagemids were produced from *E. coli* TG1 and used for screening anti-EGFR sdAbs.

Phage manipulation was performed as previously described [23]. Briefly, the *E. coli* sdAb library was infected by M13 helper phages. Phages were collected by PEG/NaCl precipitation. Immuno MaxiSorb tubes (Nunc, Rochester, NY, USA) were coated with an EGFR protein fragment located in EGFR extracellular domain III at 100, 50, 50, 25 and 25 μg/ml, respectively for the first, second, third, fourth and fifth round of screening. Phages in the sdAb library were incubated. After bound phages were eluted, TG1 was infected and cultured overnight. Colonies were scraped from the plates, and TG1 were infected with KM13 helper phages. Phages were concentrated by PEG/NaCl precipitation and used for the next round of library screening.

### Polyclonal phage ELISA

Phages derived from the library screening were checked using polyclonal phage ELISA. EGFR fragment (0.2 μg/well) or BSA as a control was used to coat wells of a 96-well plate at 4 °C overnight. After being blocked for 2 h at room temperature, phages from each round of screening were added to appropriate wells. Then, the anti-M13-HRP secondary antibody (Sino Biological, Shanghai, China) was added, and the plates were incubated. TMB (3, 3′, 5, 5′-Tetramethylbenzidine) (Beyotime Institute of Biotechnology, Haimen, China) was added. The reaction was stopped with sulfuric acid after color development. Absorbance of each well was measured at 450 nm by an automated microplate reader (Bio-RAD 680, Bio-RAD, Hercules, CA, USA).

### Monoclonal phage ELISA

After phages derived from the five rounds of screening were checked by the polyclonal phage-ELISA, *E. coli* TG1 was infected with phages from the fifth round of screening showing the highest absorbance. A total of 448 bacterial colonies were randomly picked and cultured in 96-well plates, and phage clones were derived from these bacterial clones by the infection of M13 helper phages.

For monoclonal phage ELISA, each well of 96-well plates was coated with EGFR protein fragment and blocked with 2% BSA. Eight unrelated antigens including VEGF, EndoF1, CampH, HER2, BMP2, SPB2, FGF21 and CXCR4 were included as negative controls. The plates were washed three times. The remaining steps are the same as polyclonal phage ELISA as described above. Each of these anti-EGFR sdAb clones was then sequenced, and different DNA sequences were identified by the comparison of all clones sequenced.

### Expression and purification of sdAbs

To express and purify the anti-EGFR sdAbs, the clones were amplified by PCR and ligated into the expression vector pET22b (+) (Novagen) using a forward primer (5′-GATCCATGGCCCAGGTGCAGCTGT-3′) containing a NcoI site and a reverse primer (5′-TCTGCGGCCGCGCTCGAGAC-3′) containing a NotI site. The recombinant plasmid was transformed into *E. coli* BL21 (DE3) (Novagen). Bacterium clones were randomly picked and incubated overnight at 37 °C at 220 rpm in LB medium containing ampicillin. IPTG (Sangon Biotech) was added. Culture medium was harvested, and bacterium pellet was resuspended in PBS containing PMSF (Sangon Biotech). Protein was dissolved after bacteria were broken down by the sonication. Soluble protein extract was obtained by centrifugation, and protein was purified by a Ni^+^ -NTA resin column (Sevensea Biotech). Protein was examined by 15% SDS-PAGE, and protein concentration was determined by BCA kit (Sangon Biotech).

### ELISA assay with the purified anti-EGFR sdAbs

Each well of 96-well plates was coated with the EGFR protein fragment or the seven unrelated antigens (VEGF, EndoF1, CampH, HER2, BMP2, FGF21 and CXCR4) as negative controls. Plates were washed, blocked and incubated with each of the five purified anti-EGFR sdAbs. The remaining steps are the same as polyclonal phage ELISA described above except anti-M13-HRP secondary antibody was replaced by protein A-HRP secondary antibody (Thermo Fisher).

### Western blotting

EGFR complete extracellular domain protein was purchased commercially (Shanghai Bootech BioSci. and Technol., Shanghai, China). The proteins (0.5 μg) were separated by sodium dodecyl sulfate polyacrylamide gel electrophoresis (SDS-PAGE) and transferred to a polyvinylidene fluoride (PVDF) membrane. The blots were incubated with the anti-EGFR sdAbs, followed by the incubation with protein A-HRP. The protein bands were detected by Beyo-enhanced chemiluminescence (BeyoECL) plus (Beyotime).

### Flow cytometric analysis (FACS)

Cells were harvested and resuspended in ice-cold PBS containing BSA. Anti-EGFR sdAbs or negative control sdAbs were added and incubated with cells. An anti-EGFR antibody (Santa Cruz Biotechnology, Dallas, TX, USA) was included as a positive control, and an isotype antibody (Santa Cruz Biotechnology) as a negative control. To detect the binding of the anti-EGFR sdAbs or negative control sdAbs, a protein A conjugated to fluorescein isothiocyanate (FITC) (Abcam, Cambridge, MA, USA) was used. A mouse IgG kappa binding protein conjugated to phycoerythrin (PE) (Santa Cruz Biotechnology) was used for the detection of binding of anti-EGFR antibody as a positive control and isotype antibody as a negative control. FACS Calibur (BD Biosciences) was used for the detection of fluorescence associated with the live cells, and data were analyzed by FlowJo software (BD Biosciences).

### MTT assay

For MTT assay, 5 × 10^3^ cells per well were seeded in 96-well plates and cultured in a 5% CO_2_ humidified incubator for 24 h at 37 °C. After cell medium was removed, cells were starved in serum-free medium for 4 h. Different concentrations (0, 25, 50 and 100 µg/ml) of the purified sdAbs were added, and the plates were incubated for 72 h at 37 °C. Then, 100 µl of 1 mg/ml MTT (Sigma-Aldrich) was added, and the plates were incubated for 4 h. MTT was moved, and 100 µl of DMSO (Sigma-Aldrich) was added. The plates were incubated for 10 min at 37 °C with shaking. Absorbance was measured at 570 nm by an automated microplate reader (Bio-RAD 680).

### Apoptosis assay

Apoptosis assay was performed using an Annexin V/PI apoptosis detection kit (Sangon Biotech) according to the manufacturer’s protocol. A total of 5 × 10^5^ cells were cultured overnight in 6-well plates. Cells were starved by the incubation in serum-free medium for 4 h, and the plates were washed twice with PBS. Cell medium containing 50 ng/ml of the purified sdAbs was then added. Cells were harvested after 48 h incubation, washed once with cold PBS and resuspended in 1× binding buffer at 2 × 10^6^ cells/ml. Cells were incubated with 5 μl of Annexin V-FITC and 10 μl of propidium iodide (PI) for 10–15 min at room temperature and protected from light. Cells were examined by a flow cytometry (BD Biosciences), and data were analyzed by FlowJo software (BD Biosciences).

### Cell scratch assay

The effect of the anti-EGFR sdAbs on the cell migration was evaluated by cell scratch assay. A total of 2 × 10^5^ cells were cultured in 12-well plates at 37 °C. When cell confluence reached at 90%, the cells were starved by the incubation for 4 h in serum-free medium. A single straight scratch was made across the center of the cell monolayer in each well by using a sterile 200 µl pipette tip. Plates were washed, and different concentrations (0, 25, 50,100 µg/ml) of the purified anti-EGFR sdAbs or the negative control sdAbs in 500 µl of medium containing 1% FBS (Invitrogen) was added into each well. Images were photographed by an inverted optical microscope (Nikon, Tokyo, Japan) at 100× magnification after 0 and 24 h following making a scratch on the cell monolayer. Scratch widths were measured using the Image-Pro Plus 6.0 software (Media Cybernetics, Rockville, MD, USA). Cell migration rate (%) was calculated by the formula: Lm = (L0 − Lt)/L0 × 100%, where Lm refers to the cell migration rate (%), L0 refers to scratch width at 0 h, and Lt refers to scratch width at 24 h.

### Transwell assay for detecting the cell migration and invasion

Transwell assay was used for the evaluation of effect of anti-EGFR sdAbs on cell migration. Transwells (8 µm pore size, BD Biosciences) were placed in 24-well plates. A total of 2 × 10^4^ cells in 200 µl of medium containing 1% FBS (Invitrogen) and different concentrations (0, 25, 50, or 100 µg/ml) of the purified sdAbs were added into the upper chamber of each transwell, and 600 µl of medium containing 20% FBS was added to each well of the 24-well plates. After the incubation for 24 h at 37 °C, cells remaining on the upper chamber membrane were wiped off with a cotton-tipped applicator, and the upper chambers were washed twice with PBS. Cells which migrated across the chamber membrane were fixed for 30 min with 4% paraformaldehyde (Sigma-Aldrich). Then, cells were stained with 0.1% crystal violet (Beyotime) for 30 min at room temperature. An inverted optical microscope (Nikon) was used for photographing images at 100× magnification. Cells stained with crystal violet were dissolved in 33% acetic acid solution, and absorbance at 570 nm was measured by an automated microplate reader (Bio-RAD 680).

Transwell assay was also performed to detect the cell invasion as described above except each upper chamber of transwells was coated with 60 μl of diluted matrigel (BD Biosciences) overnight at 37 °C before cells were added into transwells.

### Animal studies

All animal experiments were performed in accordance with the protocols approved by the Institutional Animal Care and Use Committee of Jinan University. Male BALB/c nude mice (4 weeks old) were purchased from Guangdong Medical Experimental Animal Center (Guangzhou, China). Mice were housed in air-filtered laminar flow cabinets with a 12 h light cycle. A total of 5 × 10^6^ A549 cells in 0.1 ml of serum-free medium were injected subcutaneously in the right flank. When tumor sizes reached approximately 100 mm^3^ on average, mice were randomly divided into six groups (five mice/group). The test reagents (10 mg sdAbs/kg or 2 mg DDP/kg) were injected intravenously once every 3 days. Tumor volumes (mm^3^) were calculated by the formula: 0.5 × (length × width^2^).

### Immunohistochemistry

Mice were sacrificed on the 24th day following the first injection of the test reagents, and tumors were removed. Tumors were fixed with formalin and embedded in paraffin. The 4 µm paraffin sections were stained by hematoxylin-eosin (HE, Beyotime).

For immunohistochemistry, 4 µm paraffin sections were incubated with each of primary antibodies (anti-Ki67, anti-CD31 and anti-caspase 3, Sigma-Aldrich) at 1:200 dilution overnight at 4 °C. The sections were washed three times with PBS for 5 min each and incubated with HRP-labeled goat anti-rat secondary antibody (Sigma-Aldrich). After the incubation for 1 h at 37 °C, the sections were washed three times with PBST for 5 min each and incubated with diaminobenzidine (DAB) chromogen (Sigma-Aldrich) for 3–5 min to show a dark brown color. The sections were photographed using an Olympus IX70 light microscope (Olympus, Tokyo, Japan), and the integrated optical density (IOD) of each image was analyzed with Image-Pro Plus analysis software (Media Cybernetics).

### Statistical analysis

Data were expressed as mean ± standard deviation (SD). All graphs were prepared with Graphpad Prism version 8.0 (Graphpad, La Jolla, CA, USA). Statistical analysis was performed using a one-way analysis of variance test (one-way ANOVA). For all statistical comparisons, *P *<* 0.05* was considered statistically significant, whereas *P *<* 0.01* was considered very significant.

## Results

### Screening a fully human sdAb phage library for anti-EGFR sdAbs

**Fig. 1:**
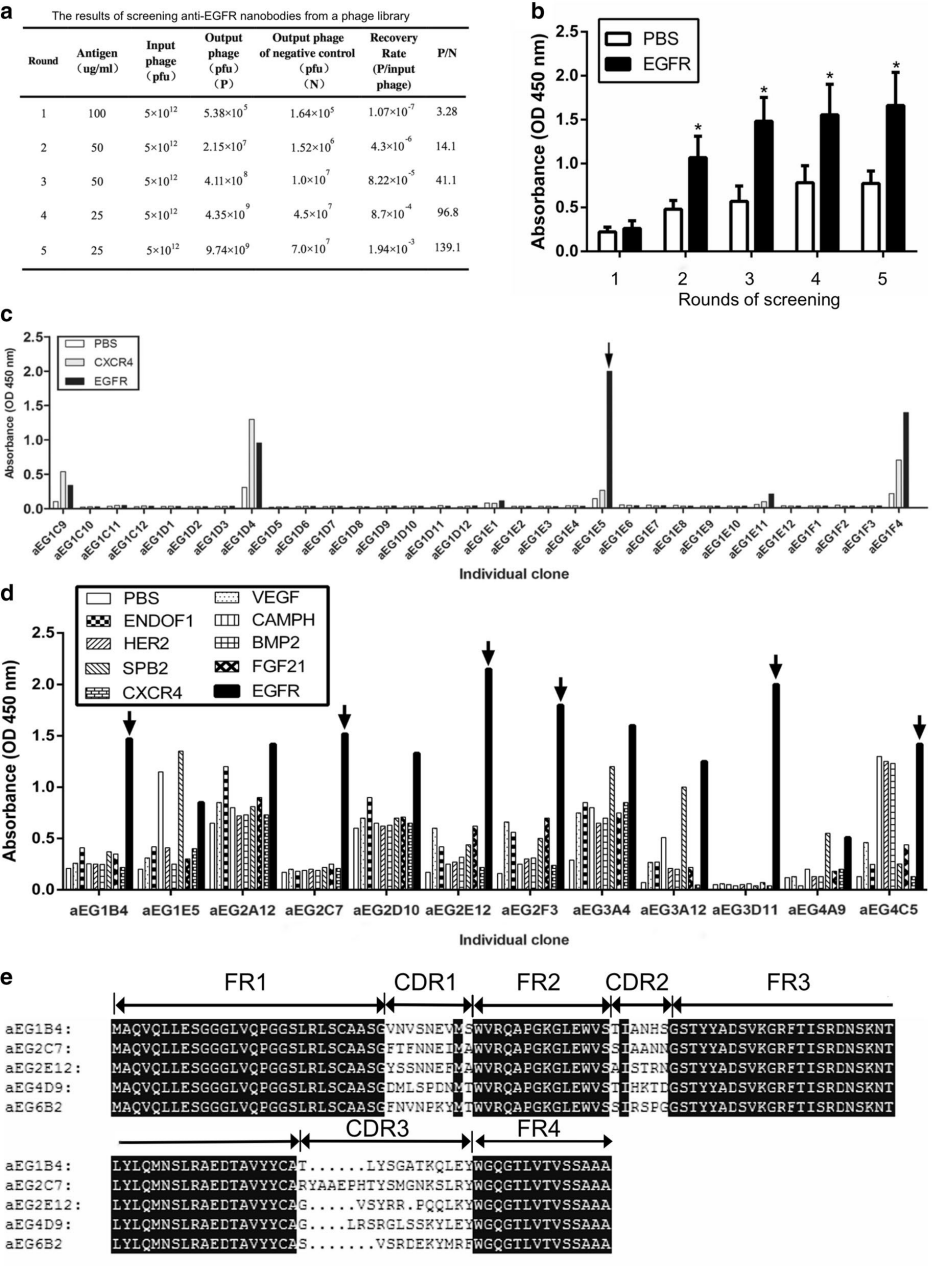
Selection of the five anti-EGFR sdAbs from a fully human sdAb phage library. **a** The P/N ratios of sdAbs derived from a fully human sdAb phage library increased along with each round of library screening. **b** The output phages derived from each round of library screening against the EGFR protein fragment were used for polyclonal phage ELISA. PBS was included as a control. Data are shown as mean ± S.D. (n = 5). *P < 0.01 vs. the respective PBS control. **c** The output phages derived from the fifth round of library screening were collected. After TG1 was infected with the phages, phage clones were randomly picked, and their binding to the EGFR protein fragment were tested by monoclonal phage ELISA. The results of representative 32 clones were shown. The arrow marked the phage clone which could specifically bind to EGFR. **d** The specific binding of EGFR to the phage clones were further examined by monoclonal phage ELISA with the EGFR protein fragment and the other eight unrelated proteins (VEGF, EndoF1, CampH, HER2, BMP2, SPB2, FGF21 and CXCR4) as negative controls. The results of representative 12 clones were shown. The arrows marked the phage clones which specifically bound to EGFR. **e** The peptide sequences of the five anti-EGFR sdAbs (aEG1B4, aEG2C7, aEG2E12, aEG4D9 and aEG6B2) were predicted from their nucleotide sequences by the DNAMAN software. Complementarity determining regions (CDR) and framework region (FR) were indicated

The phage display sdAb library was rescued by the M13 helper phage. Five rounds of library screening against EGFR fragment were carried out. The results of library screening were shown in Fig. 1a, and the enrichment ratio (P/N) increased to 139.1 after five round rounds of screening. Screened phages were tested for their binding to the EGFR fragment by the polyclonal phage ELISA. The results showed that sdAbs targeting EGFR were significantly enriched in the screening (Fig. 1b).

Subsequently, 448 phage clones were randomly picked from the fifth round of screening and tested for their binding to EGFR fragment by monoclonal phage ELISA. The results of the representative 32 clones were shown in Fig. 1c. The 24 clones showed strong binding to EGFR and were further tested by monoclonal phage ELISA by including the EGFR fragment and the other eight unrelated proteins (VEGF, EndoF1, CampH, HER2, BMP2, SPB2, FGF21 and CXCR4) as negative controls. The results showed that 13 phage clones could specially bind to EGFR fragment and not to the other eight unrelated proteins (Fig. 1d). These 13 phage clones were DNA-sequenced, and the five different sdAbs were obtained, including aEG1B4 (accession number: LR743560), aEG2C7 (accession number: LR743561), aEG2E12 (accession number: LR743562), aEG4D9 (accession number: LR743563) and aEG6B2 (accession number: LR743564) (Fig. 1e). These five sdAbs share the same four framework regions (FR1–4) and have different CDR1–3.

### Expression and characterization of the five anti-EGFR sdAbs

**Fig. 2:**
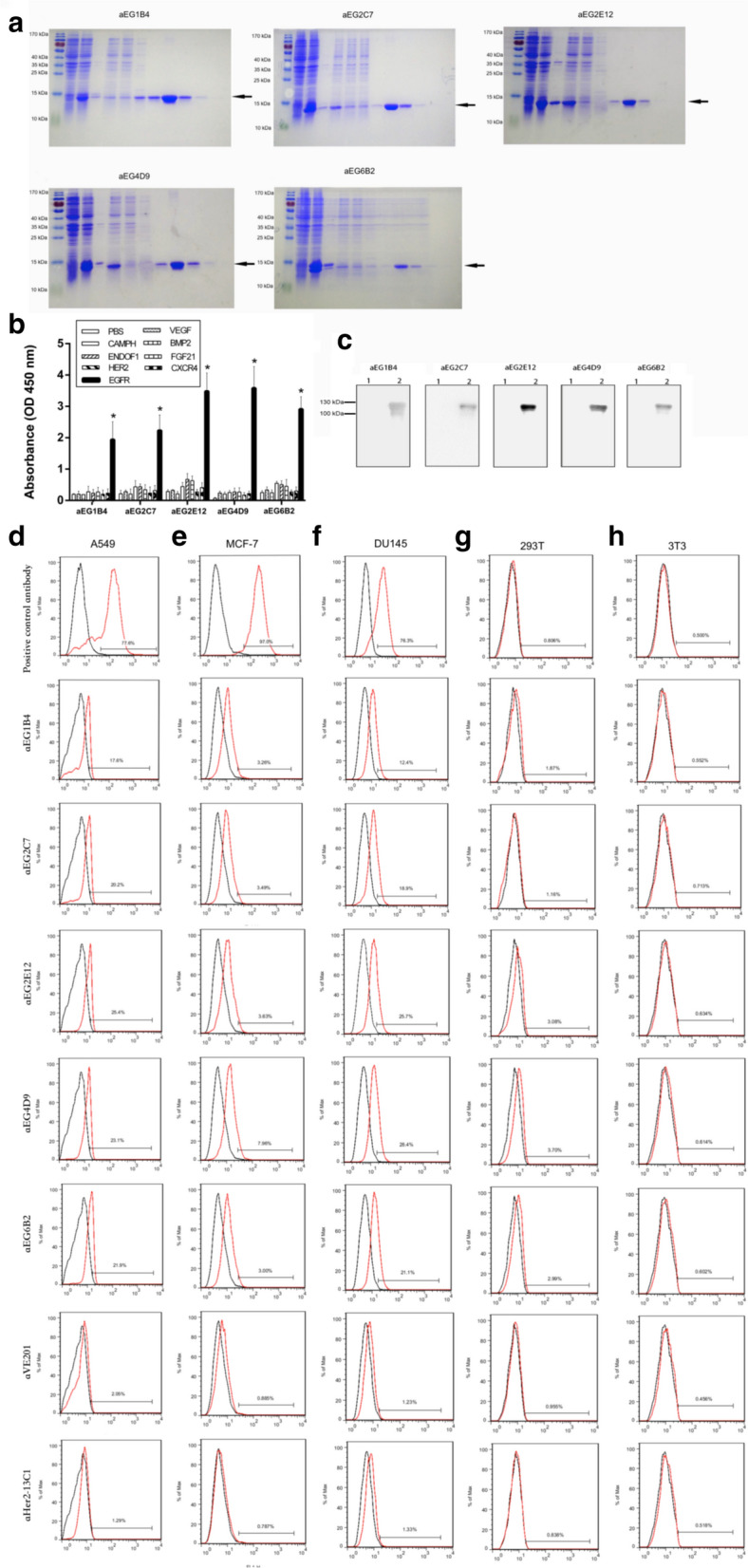
Purification and characterization of the five anti-EGFR sdAbs. **a** SDS-PAGE was performed to examine the five anti-EGFR sdAbs (aEG1B4, aEG2C7, aEG2E12, aEG4D9 and aEG6B2). Left lane, protein marker; lane 1, uninduced total bacterial protein; lane 2, induced total bacterial protein; lane 3, insoluble fraction after bacterial breakage; lane 4, soluble fraction after bacterial breakage; lane 5, flow-through fraction; lane 6, wash buffer; lane 7-11, fractions collected from the protein purification column. The sdAb positions were marked by the arrows. **b** The specific binding of the five purified anti-EGFR sdAbs to the EGFR protein fragment was evaluated with ELISA. EGFR protein fragment and the other seven unrelated proteins (VEGF, EndoF1, CampH, HER2, BMP2, FGF21 and CXCR4) as negative controls were included. **c** Western blot analysis was performed with the five purified anti-EGFR sdAbs against the EGFR complete extracellular fragment purchased commercially (lane 2) and PBS as a control (lane 1). The specific binding of the five purified anti-EGFR sdAbs to cancer cells was examined by flow cytometric analysis (**d**–**h**). The three human cancer cell lines A549 (**d**), MCF-7 (**e**) and DU145 (**f**) were tested. The two cell lines 293T (**g**) and 3T3 (**h**) were included as negative controls. Black curves showed the background staining with an isotype control primary antibody or no primary antibody. Red curves showed the staining with anti-EGFR antibody purchased commercially as a positive control, the five purified anti-EGFR sdAbs (aEG1B4, aEG2C7, aEG2E12, aEG4D9 and aEG6B2), or the two purified sdAbs (aVE201 and aHer2-13C1) as negative controls

For expression of these five sdAbs, the coding sequences of the sdAbs were sub-cloned into the pET-22b vector under the T7 promoter, and the plasmids were transformed into *E. coli* BL21 (DE3). The expression of soluble sdAb proteins was induced by IPTG, and the proteins were purified by a Ni^+^ -NTA resin column. The eluted proteins were separated on SDS-PAGE gel and visualized by coomassie brilliant blue staining, and each purified sdAb showed a single band marked by an arrow (Fig. 2a).

The sdAbs were tested by ELISA for their binding to EGFR fragment and the seven unrelated proteins (VEGF, EndoF1, CampH, HER2, BMP2, FGF21 and CXCR4) as negative controls. The results showed that all of these five sdAbs specifically bound to EGFR fragment and not the seven unrelated proteins, and two (aEG2E12 and aEG4D9) of them showed the higher binding than the others (Fig. 2b). These sdAbs were further tested by Western blotting for their binding to EGFR complete extracellular fragment purchased commercially, and the results showed that all of the five sdAbs could bind to EGFR complete extracellular fragment, and two (aEG2E12 and aEG4D9) of them showed higher signal than the others (Fig. 2c) and were chosen for further testing by animal studies.

In addition, the sdAbs were tested by flow cytometric analysis for their binding to cancer cells. The results showed that the five anti-EGFR sdAbs specifically bound to the human cancer cells A549, MCF-7 and DU145 and not the cells 293T and 3T3 as negative controls (Fig. 2d–h). The anti-EGFR antibody purchased commercially was included as a positive control and showed the results similar to the five anti-EGFR sdAbs. Two sdAbs (aVE201 and aHer2-13C1) were included as negative controls. These two sdAbs (aVE201 and aHer2-13C1) were isolated previously in our laboratory in a different study from the same human DAb library (previously unpublished data) and could not bind to EGFR. Their proteins were purified with the same method as the five anti-EGFR sdAbs.

### The anti-EGFR sdAbs inhibited cancer cell proliferation and increased their apoptosis

**Fig. 3:**
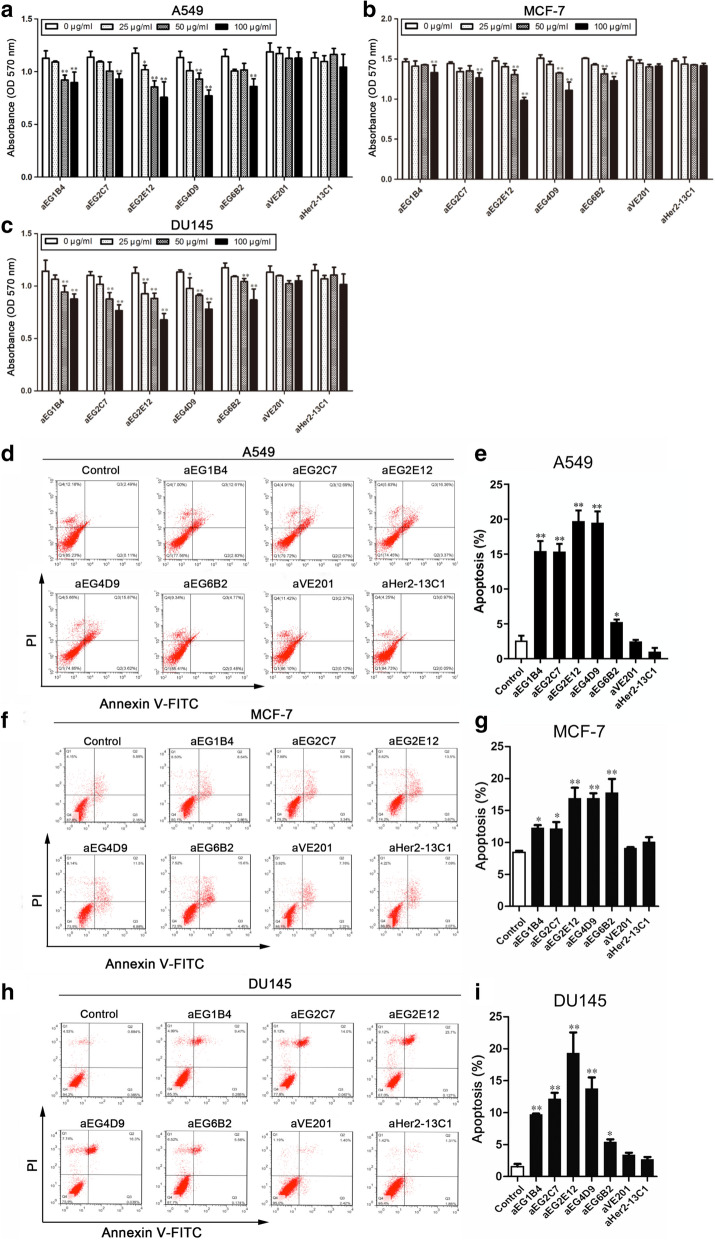
Effect of the five anti-EGFR sdAbs on inhibiting cell proliferation and promoting cell apoptosis. The inhibition of cell proliferation by the five anti-EGFR sdAbs (aEG1B4, aEG2C7, aEG2E12, aEG4D9 and aEG6B2) was tested by MTT assay on cancer cells A549 (**a**), MCF-7 (**b**) and DU145 (**c**). Cells were treated with different concentrations (0, 25, 50 and 100 µg/ml) of the anti-EGFR sdAbs. Two sdAbs (aVE201 and aHer2-13C1) were included as negative controls. Effect of the five anti-EGFR sdAbs on apoptosis was evaluated by apoptosis assay with cancer cells A549 (**d**, **e**), MCF-7 (**f**, **g**) and DU145 (**h**, **i**). Cells were incubated with 50 µg/ml of the anti-EGFR sdAbs. Annexin V-FITC was used to determine the percentage of cells undergoing apoptosis at an early stage, and propidium iodide (PI) was used to distinguish between viable and nonviable cells. Two sdAbs (aVE201 and aHer2-13C1) were included as negative controls. Data are shown as mean ± S.D. (n = 5). For **a**–**c**, *P < 0.05 and **P < 0.01 vs. the respective control (0 µg/ml); for **e**, **g** and **i**, *P < 0.05 and **P < 0.01 vs. PBS control

The sdAbs were tested by MTT assay for their effect on the proliferation of cancer cells A549, MCF-7 and DU145. The results showed that the cell proliferation was significantly inhibited by all the five sdAbs in a dose-dependent manner (Fig. 3a–c). More inhibition was seen at the higher sdAb concentrations. Two sdAbs (aVE201 and aHer2-13C1) were included as negative controls and showed no inhibition on cell proliferation at all the concentrations.

Effect of the sdAbs on cancer cell apoptosis was also investigated by apoptosis assay. The results showed that all the five anti-EGFR sdAbs significantly increased cancer cell apoptosis at the concentration of 50 μg/ml (Fig. 3d–i). The two sdAbs (aVE201 and aHer2-13C1) were included as negative controls and did not increase cell apoptosis of all the three cancer cell lines.

### Anti-EGFR sdAbs inhibited cancer cell migration and invasion

**Fig. 4:**
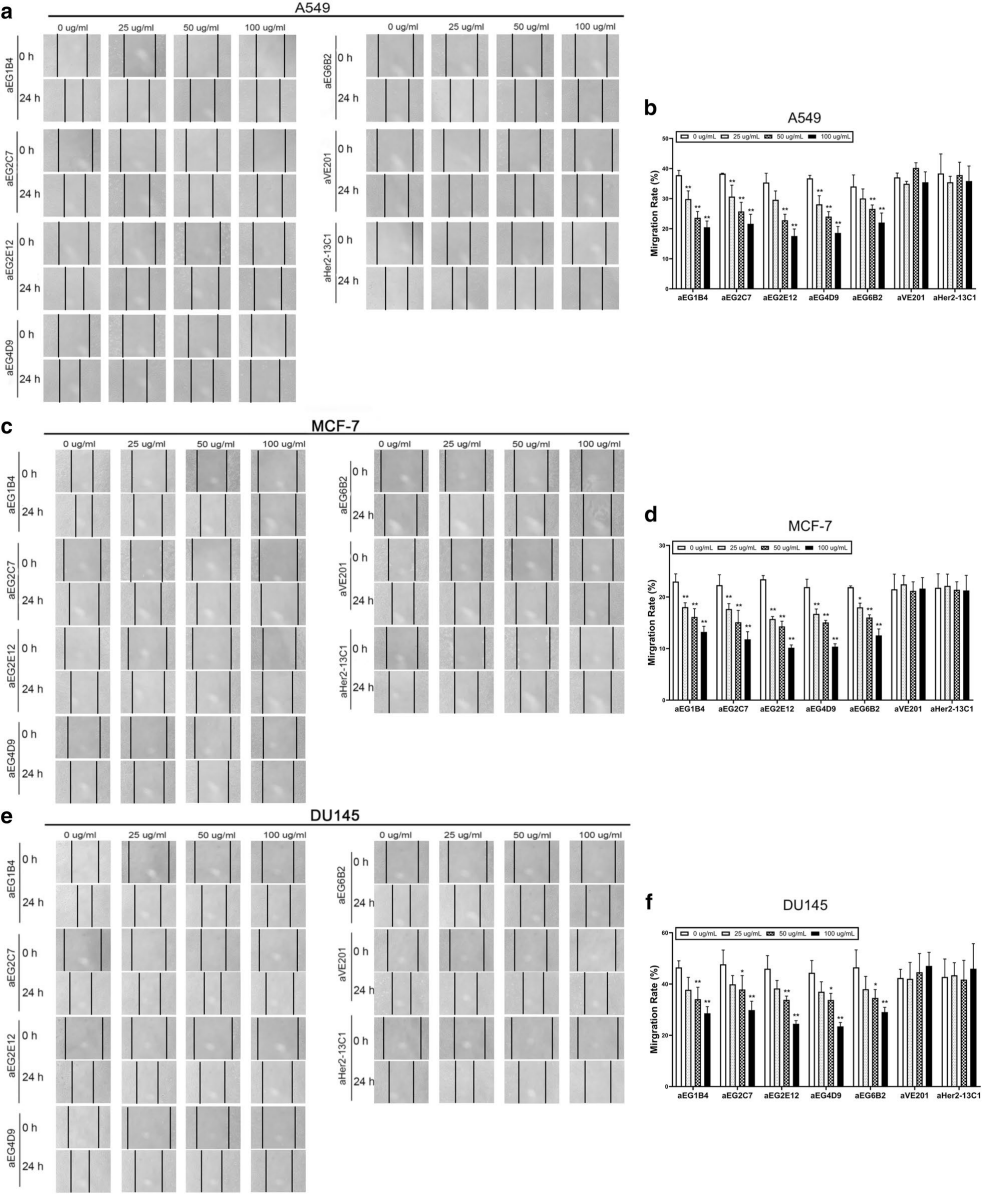
Effect of the five anti-EGFR sdAbs on inhibiting cancer cell migration by cell scratch assay. Cells were incubated for 24 h with the five anti-EGFR sdAbs (aEG1B4, aEG2C7, aEG2E12, aEG4D9 and aEG6B2) at different concentrations (0, 25, 50 and 100 µg/ml). Two sdAbs (aVE201 and aHer2-13C1) were included as negative controls. Images were photographed after 0 and 24 h following making a scratch on a cell monolayer. The scratch widths were measured by Image-Pro Plus 6.0 software. **a**, **b** A549 cells; **c**, **d** MCF-7 cells; **e**, **f** DU145 cells. Data are shown as mean ± S.D (n = 5). *P < 0.05 and **P < 0.01 vs. the respective control (0 µg/ml)

Effect of the sdAbs on cancer cells migration was investigated by the cell scratch assay. The results showed that all the five sdAbs could inhibit the migration of all the three cancer cell lines (Fig. 4). The inhibition was generally concentration-dependent. The two sdAbs (aVE201 and aHer2-13C1) were included as negative controls and showed no inhibition on cell migration.

**Fig. 5:**
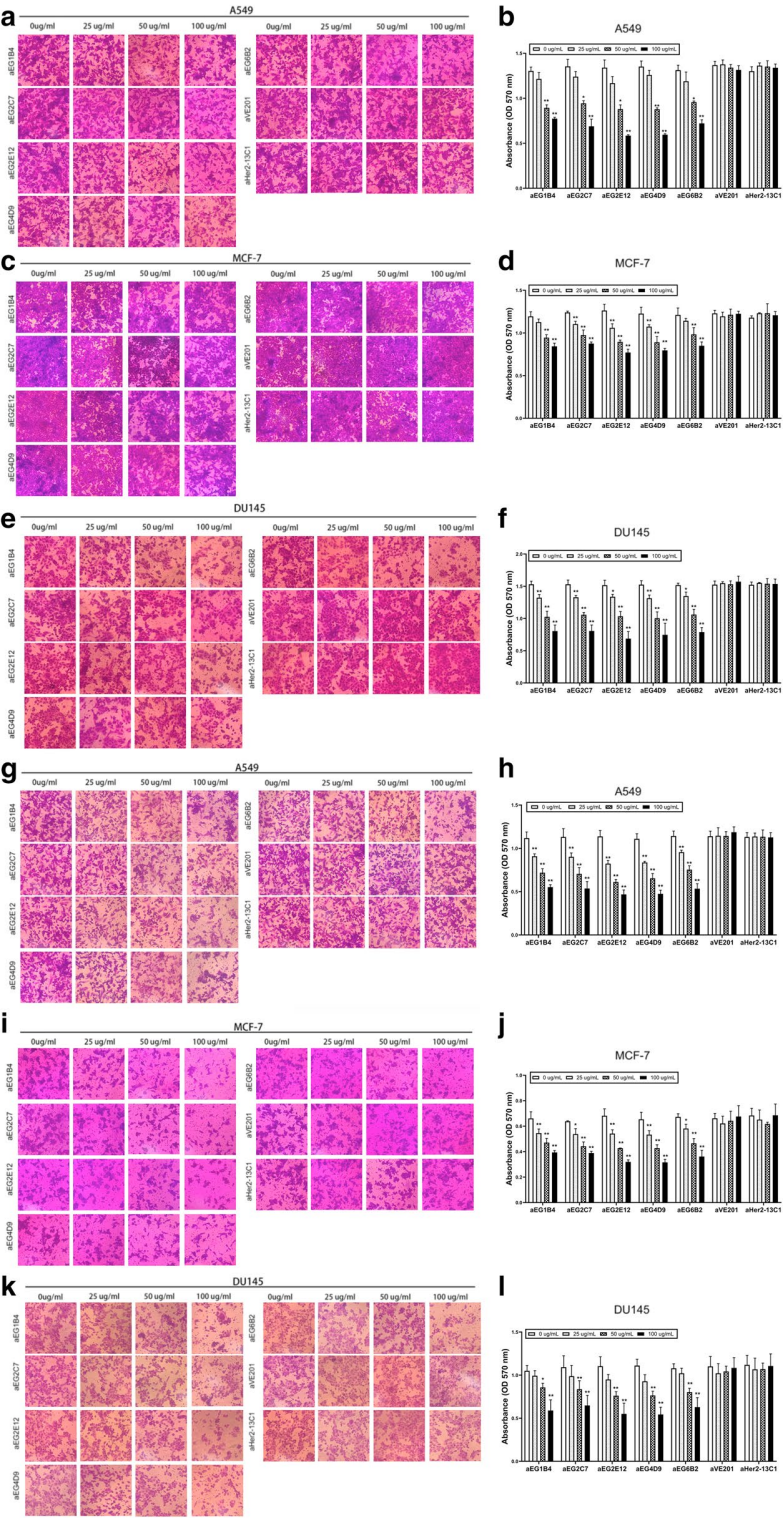
Effect of the five anti-EGFR sdAbs on inhibiting cell migration and invasion by the transwell assay. **a**–**f** The inhibition of cancer cell migration by anti-EGFR sdAbs was shown. Cells in the medium containing 1% FBS and the five purified anti-EGFR sdAbs (aEG1B4, aEG2C7, aEG2E12, aEG4D9 and aEG6B2) at different concentrations (0, 25, 50 or 100 µg/ml) were seeded into the upper chambers of transwells. Two sdAbs (aVE201 and aHer2-13C1) were included as negative controls. After 24 h of incubation, cells which migrated to the bottom chamber membrane were stained with 0.1% crystal violet. Images were photographed. Crystal violet on the bottom chamber membrane was dissolved in 33% acetic acid solution, and absorbance at 570 nm was measured. **a**, **b** A549 cells; **c**, **d** MCF-7 cells; **e**, **f** DU145 cells. Detecting cell invasion (**g**–**l**) was performed as described above except each upper chamber of transwells was coated with 60 μl of diluted matrix gel overnight at 37 °C before cells and sdAbs were added into transwells. **g**, **h** A549 cells; **i**, **j** MCF-7 cells; **k**, **l** DU145 cells. Data are shown as mean ± S.D. (n = 5). *P < 0.05 and **P < 0.01 vs. the respective control (0 µg/ml)

Effect of the five sdAbs on cancer cell migration and invasion was also examined by the transwell assay. Data showed that all the five anti-EGFR sdAbs could inhibit the migration (Fig. 5a–f) and invasion (Fig. 5g–l) of all the three cancer cell lines in a dose-dependent manner.

### Two sdAbs inhibited tumor growth in vivo

**Fig. 6:**
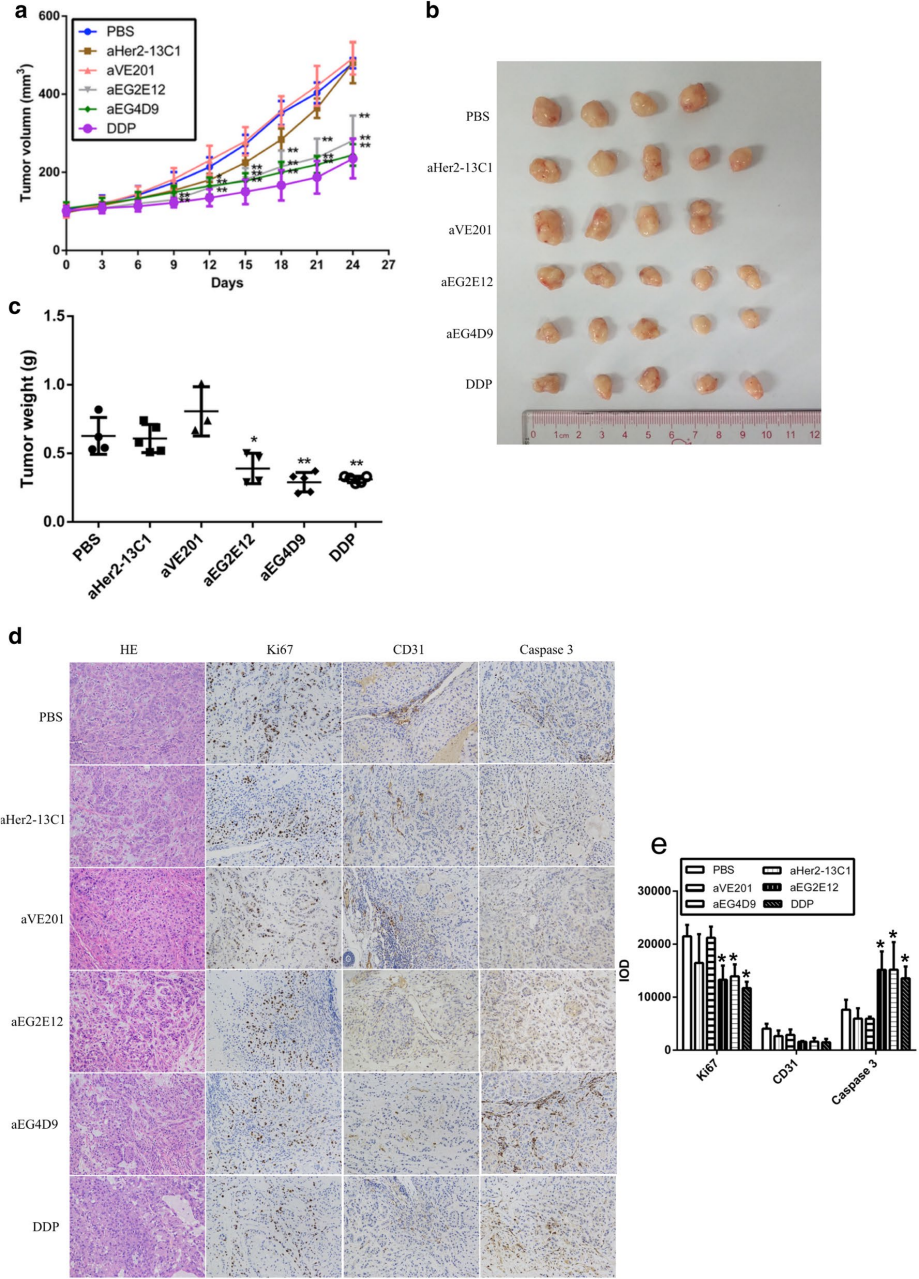
Inhibition of A549 xenograft growth in BALB/c nude mice by anti-EGFR sdAbs. A total of 5 × 10^6^ A549 cells were injected into the right flank of each mouse. SdAbs were injected after tumor sizes reached to about 100 mm^3^. **a** Mice were injected with 10 mg/kg anti-EGFR sdAbs (aEG2E12 and aEG4D9) every 3 days. PBS, DDP (2 mg/kg) and two sdAbs (aVE201 and aHer2-13C1, 10 mg/kg) were included as controls. **b**, **c** Mice were sacrificed on the 24th day following the first injections of the test reagents, and tumors were removed, photographed and weighted. **d** Tumor sections were stained by HE or examined with anti-Ki67, anti-CD31 and anti-caspase 3 antibodies. **e** Integral optical density (IOD) was calculated from **d** by Image-Pro Plus analysis software. Data are shown as mean ± S.D. (n = 4). *P < 0.05 and **P < 0.01 vs. the respective PBS control

Cancer cell A549 mouse xenograft model was used to validate the anti-tumor potential of the sdAbs in vivo. The two sdAbs aEG2E12 and aEG4D9 were selected for this study and the two sdAbs (aVE201 and aHer2-13C1) were included as negative controls. When the tumors reached an average volume of 100 mm^3^, BALB/c nude mice were injected intravenously with PBS, 2 mg/kg of DDP as positive control, 10 mg/kg of anti-EGFR sdAbs (aEG2E12 and aEG4D9) and negative control sdAbs (aVE201 and aHer2-13C1) every 3 days. The results showed that the two anti-EGFR sdAbs significantly inhibited the tumor growth compared with the PBS and the two sdAbs as negative controls (Fig. 6a–c). The inhibition of tumor growth by the two anti-EGFR sdAbs was comparable with DDP.

### Two sdAbs inhibited cancer cell proliferation and increased their apoptosis in vivo

To preliminarily study the mechanisms how the anti-EGFR sdAbs inhibited cancer cell growth in vivo, immunohistochemistry was performed with the tumors taken from the mouse tumor xenograft model. Tumor cell proliferation was detected by anti-Ki67 antibody, tumor angiogenesis by anti-CD31 antibody, and tumor cell apoptosis by anti-caspase 3 antibody. The results demonstrated that the two anti-EGFR sdAbs and DDP inhibited tumor cell proliferation, increased tumor cell apoptosis and did not show effect on tumor angiogenesis (Fig. 6d, e). On the other hand, PBS and the two sdAbs as negative controls had no effect on tumor cell proliferation, angiogenesis and apoptosis.

## Discussion

Chemotherapy and radiotherapy are commonly used for cancer therapy, but they usually result in low cure rate and cause serious side effects. New cancer therapies with reduced toxicity and elevated efficacy have been pursued. Although mAbs have been successful for cancer therapy, they have many disadvantages including low efficacy, high side-effect and high cost, and these limit their applications. The mAb major disadvantages are described as follows.

First, mAbs are very large in size and are difficult to penetrate into tumor tissues, so that the mAb efficacy is limited. In solid tumors, the cancer cells are in the increased state of hypoxia and interstitial fluid pressure due to the broken-down of homeostatic regulation [24]. The tumor extracellular matrix (ECM) hinders the movement of mAb into the tumor tissues [25]. Thus, some attempts have been made to develop smaller antibodies, which still preserve the specificity and affinity of the conventional mAbs and have lower immunogenicity to decrease the immune rejection. In this study, the five anti-EGFR sdAbs of only 15 kDa were identified and could specifically bind to EGFR and the human cancer cells. They could significantly inhibit cancer cell proliferation in vitro and tumor growth in vivo.

Second, mAbs for cancer therapy are normally isolated from mouse and are mouse antibodies against human proteins. Mouse antibody is recognized as a foreign antigen in human and can be rejected by human immune system, which can result in rapid mouse antibody clearance, low anti-tumor efficacy or hypersensitivity reactions in human body [12]. As the development of genetic engineering, humanized antibody or chimeric antibody were developed to reduce the antigenicity of mouse antibody [13]. Even though immunogenicity of the mouse antibody may be reduced, it cannot be completely eliminated. Moreover, the potential antibody immunogenicity may be increased by mAb formulation, aggregation and glycosylation and a cell line used to express the antibody. For instance, cetuximab produced in NS0 cells contained alpha-galactose carbohydrate that caused hypersensitivity reaction in some allergic individuals [26].

Third, because human sdAb is small, it can access a hidden epitope that is not accessible to the large-size conventional mAb [20, 21].

Fourth, mAbs need to be expressed in mammalian cells (usually CHO cells), and the manufacturing process is very expensive and leads to the high cost of mAb medicines. On the other hand, human sdAb of about 15 kDa consists of only the variable domain of the human antibody heavy chain (VH) and can be expressed in bacteria. The cost of sdAb production is much lower than mAb [27]. In this study, the five anti-EGFR sdAbs were expressed in *E. coli* BL21 and purified by Ni^+^-NTA chromatography. They could bind to EGFR and showed good anti-tumor activity in vitro and in vivo. Therefore, human sdAb is an ideal alternative for the conventional mAbs for cancer therapy.

Some cancer-specific proteins were identified. EGFR overexpression in certain cancer cells promoted tumor development and metastasis, and EGFR is the marker for poor cancer prognosis [28–30]. It also promoted tumor angiogenesis via inducing the expression of vascular endothelial growth factors (VEGF). Clinical application showed that the anti-EGFR antibodies were effective for cancer therapy, and EGFR was an important anti-cancer target [31]. Anti-EGFR mAbs and small molecule tyrosine kinase inhibitors (TKIs) targeting EGFR were approved by FDA for cancer therapy. Anti-EGFR mAbs including panitumumab, cetuximab and nimotuzumab were approved for treatment of NSCLC and colorectal cancer [15, 16, 32].

The EGFR extracellular domain III is critical for EGF binding. Structural analysis of the EGFR-GC1118 (an anti-EGFR mAb) crystal complex revealed that GC1118 recognizes linear and discrete N-terminal epitopes of domain III of EGFR [33]. GC1118 exhibited potent inhibitory activity against high-affinity EGFR ligands in terms of EGFR binding, triggering EGFR signaling and proliferation [33]. The binding epitopes of two anti-EGFR mAbs approved by FDA (cetuximab and panitumumab) overlap with the EGF-binding site on the EGFR domain III [33]. Studies showed that these two antibodies could inhibit EGFR downstream pathway signalling, thus blocking the proliferation, migration and invasion of tumor cells by competing binding to EGFR with EGF [34]. Therefore, in this study, we chose an EGFR protein fragment located in the EGFR extracellular domain III for screening anti-EGFR sdAbs from a sdAb phage library.

Previous studies showed that EGFR could induce intracellular tyrosine phosphorylation. Studies also indicated that EGF/EGFR signaling pathway was interacted with EGFR/ERK, EGFR/STAT3 and EGFR/mTOR signaling pathways [3, 4]. These pathways are associated with promoting tumor cell proliferation, vitality, migration and invasion, tumorigenesis, tumor progression and metastasis and tumor angiogenesis in the tumor microenvironment [5, 6]. In this study, the five anti-EGFR sdAbs inhibited cancer cell proliferation in vitro and tumor growth in vivo. Binding of the anti-EGFR sdAbs to the EGFR extracellular domain may prevent EGF from binding to EGFR, or homophilic cancer cell–cell adhesion through cell surface EGFR interaction. Further study is required to examine the mechanisms how these anti-EGFR sdAbs inhibit cancer cell growth.

SdAb can be modified to increase its efficacy. For example, bi-specific sdAb can be generated by fusing two different sdAbs targeting EGFR antigen and a second antigen, respectively. The second antigen can include CD3, which is a T cell co-receptor and can activate T cells or CD44, which is a multifunctional cell surface molecule involved in cell proliferation, differentiation and migration and angiogenesis [35, 36]. The anti-EGFR sdAbs can be fused to a toxic molecule to form an antibody-drug conjugate (ADC) to strengthen cancer cell killing [37]. A toxic molecule can include doxorubicin (DOX) and urease enzyme [38]. The anti-EGFR sdAb can also be fused to a tumor penetrating peptide to enhance its penetration into tumor tissue and increase its efficacy for cancer therapy [39].

## Conclusions

An EGFR protein fragment located at its extracellular domain III was chosen for screening a fully human sdAb library by phage display. Five anti-EGFR sdAbs were obtained, and they specifically bound to both an EGFR protein fragment and the EGFR complete extracellular domain purchased commercially. They also bound to the human cancer cells A549, DU145 and MCF-7. They could inhibit cancer cell proliferation, migration and invasion and induce cancer cell apoptosis. Furthermore, two anti-EGFR sdAbs (aEG2E12 and aEG4D9) were tested for their anti-tumor effect in vivo, and inhibited tumor growth in a lung cancer mouse model. This study demonstrates that these anti-EGFR sdAbs may become good alternative of mAbs for cancer therapy.

## Data Availability

The datasets generated for this study can be found in the European Nucleotide Archive (https://www.ebi.ac.uk/ena/data/view/LR743560, https://www.ebi.ac.uk/ena/data/view/LR743561, https://www.ebi.ac.uk/ena/data/view/LR743562, https://www.ebi.ac.uk/ena/data/view/LR743563, and https://www.ebi.ac.uk/ena/data/view/LR743564) using the accession numbers LR743560, LR743561, LR743562, LR743563 and LR743564.

## Abbreviations

SdAb: Single-domain antibody
DDP: Cis-platinum
EGFR: Epidermal growth factor receptor
ECD: Extracellular domain
ICD: Intracellular domain
EGFR-ECD: EGFR-extracellular domain
EGFR-ICD: EGFR-intracellular domain
mAbs: Monoclonal antibodies
scFv: Single-chain variable fragment
VH: Variable domains of the mAb heavy chain
VL: Variable domains of the mAb light chain
sdAb: Single-domain antibody
Protein A-HRP: Protein A-horseradish peroxidase conjugate
IPTG: Isopropyl-β-d-thiogalactopyranoside
PMSF: Phenylmethylsulfonyl fluoride
DMSO: Dimethyl sulfoxide
FBS: Fetal bovine serum
Dab: Domain antibody library
FACS: Flow cytometric analysis
CDR: Complementarity determining region
SDS-PAGE: Sodium dodecyl sulfate polyacrylamide gel electrophoresis
PE: Phycoerythrin
HE: Hematoxylin–eosin
VEGF: Vascular endothelial growth factors
TKIs: Tyrosine kinase inhibitors
ADC: Antibody-drug conjugate
